# Researching on the fine structure and admixture of the worldwide chicken population reveal connections between populations and important events in breeding history

**DOI:** 10.1111/eva.13241

**Published:** 2021-05-05

**Authors:** Ying Guo, Jen‐Hsiang Ou, Yanjun Zan, Yuzhe Wang, Huifang Li, Chunhong Zhu, Kuanwei Chen, Xin Zhou, Xiaoxiang Hu, Örjan Carlborg

**Affiliations:** ^1^ State Key Laboratory for Agro‐Biotechnology China Agricultural University Beijing China; ^2^ Beijing Advanced Innovation Center for Food Nutrition and Human Health China Agricultural University Beijing China; ^3^ Department of Medical Biochemistry and Microbiology Uppsala University Uppsala Sweden; ^4^ Jiangsu Institute of Poultry Science Yangzhou China; ^5^ National Engineering Laboratory for Animal Breeding China Agricultural University Beijing China

**Keywords:** admixture, Asian breeds, chickens, genomic structure, selection

## Abstract

Here, we have evaluated the general genomic structure and diversity and studied the divergence resulting from selection and historical admixture events for a collection of worldwide chicken breeds. In total, 636 genomes (43 populations) were sequenced from chickens of American, Chinese, Indonesian, and European origin. Evaluated populations included wild junglefowl, rural indigenous chickens, breeds that have been widely used to improve modern western poultry populations and current commercial stocks bred for efficient meat and egg production. In‐depth characterizations of the genome structure and genomic relationships among these populations were performed, and population admixture events were investigated. In addition, the genomic architectures of several domestication traits and central documented events in the recent breeding history were explored. Our results provide detailed insights into the contributions from population admixture events described in the historical literature to the genomic variation in the domestic chicken. In particular, we find that the genomes of modern chicken stocks used for meat production both in eastern (Asia) and western (Europe/US) agriculture are dominated by contributions from heavy Asian breeds. Further, by exploring the link between genomic selective divergence and pigmentation, connections to functional genes feather coloring were confirmed.

## 1 INTRODUCTION

**FIGURE 1:**
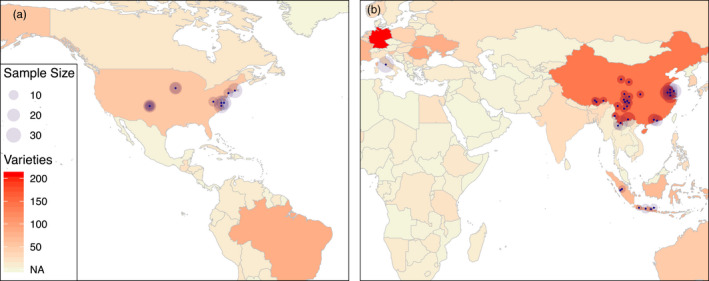
Number of recorded chicken varieties in regions of the world and the distribution of the sampled populations across these. The colors on the map (a, b) illustrate the documented chicken diversity in each region (data from http://www.fao.org/dad‐is/browse‐by‐country‐and‐species/en/). The blue dots represent the geographic locations where stocks included in this study were sampled, with sizes of the circles illustrating the numbers of individuals sampled from each population

The chicken is one of the major agricultural species, and the poultry industry today produces more than 120 million tons of meat and over 1.2 trillion eggs each year (FAO, 2020). Its domestication started about 10,000 year ago (International Chicken Polymorphism Map, 2004; Xiang et al., 2014), likely following a commensal pathway where the initial interactions were an adaptation of wild junglefowl to human‐created niches, followed by an over time increased human intent to keep, control, intensively breed and ultimately commercialize the use of chicken in agriculture (Zeder, 2012). During this process, hundreds of chicken breeds have been established across the world (Figure 1a,b), ranging from extensively kept indigenous breeds to highly specialized chicken stocks used in industrial‐scale egg (layers) or meat (broilers) production.

Earlier genomics‐based population genetics studies in livestock and nonmodel organisms have explored population differences, inferred demographic histories, and quantified admixture to provide deeper insights to the origin, evolution, and domestication of a range of species (Beichman et al., 2018; Guo et al., 2019; Lawal et al., 2020; Wang et al., 2020). Large‐scale sequencing and statistical analyses of the data are no longer the major barrier for such analyses, but rather the collection of suitable samples.

The overall aim of this study was to evaluate the population genetics of the domestic chicken with a particular focus on populations from Asia, which is the origin of the domestic chicken, and their contributions to the current worldwide population. To achieve this, extensive sampling was performed across Asia and whole‐genome re‐sequencing data were generated for chicken populations from various geographic origins and stages of domestication including wild, indigenous, intensely selected, and commercialized populations (Table S1). These data were used to evaluate the genomic diversity among the sampled chicken populations and breeds to better understand the origin of existing variation and their potential connections to various morphological domestication traits. Selection signatures in domestic stocks resulting from past breeding processes for production traits, and traits related to regional adaptation, could be compared with patterns resulting from admixture events and relationships among populations could then be used to make demographic inferences for comparisons with historical description of population dynamics and crossings. These analyses confirmed known, and suggested new, relationships and admixture events between populations within Asia and across continents. Overall, our results were in agreement with available historical records about the origin and ancestry of key breeds used in the breeding of current stocks used for commercial egg and meat production.

## 2 METHODS

### 2.1 Samples and data

The current study uses both publicly available (Guo et al., 2016, 2019; Li et al., 2017; Ulfah et al., 2016; Wang et al., 2015) and newly generated whole‐genome sequencing data (Figure 1a,b). The new sequence data (*n* = 341 individuals from 12 breeds) were generated from libraries prepared using the NeoPrep Library Prep System (Illumina) and sequenced to, on average, 8× genome coverage by paired end (2×150 bp) sequencing on the IIlumina Hiseq X Ten system at CLOUD HEALTH. Table S1 provides detailed information on all the 43 populations used. The generated sequence dataset has been uploaded to NCBI with BioProject numbers PRJNA597842, PRJNA547951, and PRJNA552722.

### 2.2 Data management

For each sample, variant calling was performed using *GATK* (McKenna et al., 2010) following the best practice pipeline. The sequence reads were mapped to the ICGSC Gallus_gallus‐5.0 reference genome (Nov. 2011) using *BWA*‐*MEM* (version 0.7.17) (Li & Durbin, 2009). The alignment results were stored in Binary Alignment/Map (BAM) format using the *SAMtools* (version 1.3.1) package (Li et al., 2009). Adding read groups, sorting read pairs, marking duplicate reads, building bam index, and validating bam files were done using the *Picard* tools v2.20.4 (http://broadinstitute.github.io/picard/). Local realignment, base quality score recalibration, and joint variant calling on all the samples were done using the *IndelRealigner*, *BaseRecalibrator*, and *HaplotypeCaller* from *GATK* (version 3.7). Variant filtering was implemented for each autosomal chromosome individually. Small indels and variants with more than 2 alleles were removed, and then, low mapping quality variants that did not meet the criteria (genotypes called >0.5, minor allele count >3, minimum quality score >20) were filtered out using *VCFtools* (Danecek et al., 2011). Genotype phasing of markers was done using *Beagle* 4.0 (Browning & Browning, 2007). After all the above steps, a pruned PED, MAP, and GENO format files were produced for downstream analyses.

### 2.3 Basic population genetic analysis

*PopLDdecay* 3.31 (Zhang et al., 2019), which is a fast tool using the VCF files as input, was used to calculate the LD decay statistics. The result was plotted as pairwise *r* ^2^ values against the physical distance. *F*_st_ statistics were calculated using *VCFtools*. The *F*_st_ window size chosen was 20 kb, and the *F*_st_ window step size was 10 kb. *F*_st_ differences were compared groupwise for traits where breeds were clustered as “cases” and “controls.” Haplotype centric detection of selection based on population differentiation was performed using *hapFLK* for the recessive white, dominant white, and black feather loci (Fariello et al., 2013). Comparisons were done also using the “cases” and “controls” populations mentioned below. Haplotypes were plotted using R with the phased haplotype files as input. The breeds were grouped as “cases” and “controls” using the following coat color classifications. The dominant white group of breeds were as follows: White Leghorn and Cobb. The recessive white group of breeds included the Recessive White, the Virginia BW lines (HWS and LWS), and Silkie. The black group of breeds included Emei Black, Muchuan Black Bone, Tianfu Black Bone, Sumatras, Langshans, Black Cochin, and Java. The yellow‐feathered group of breeds included Liyang, Buff Cochin, Huiyang Beard, and Rhode Island Red. Run of homozygosity analyses were performed using *BCFtools*/*ROH* (version 1.10.2) which is based on a hidden Markov model (Narasimhan et al., 2016). The mean number of ROH and SROH (sum total length of ROH) was then calculated (Table S2), and figures were plotted in R.

### 2.4 Population structure analysis

*Admixture* (Alexander et al., 2009) was used to estimate the ancestry of the breeds. Several K values (assumed number of ancestral populations) were evaluated in the analysis, and a cross‐validation procedure for K was used to provide a sensible modeling choice. Principal component analysis (PCA) was performed on the samples from all breeds except the Green Junglefowl (GJF) using the *EIGENSOFT* (Patterson et al., 2006) package. The population structure, clustering of samples, and admixture events dating were also investigated using *Finestructure* (Lawson et al., 2012) and *Globetrotter* (Hellenthal et al., 2014) following the standard procedures described in their respective manuals.

### 2.5 Test for gene flow

To test for possible gene flow between the populations, the ABBA‐BABA statistics (Green et al., 2010) (also called the D statistics) were used to test for introgression using genome‐scale SNP data. The Red Junglefowl was specified as the outgroup in the analysis. The standard deviation of D was computed using a block jackknife procedure. The D statistics, standard deviation of D, standard error, and Z score were all computed in R.

## 3 RESULTS

We have studied the genomic variation in a large worldwide collection of the domestic chicken breeds in detail. The general genomic structure and diversity among the breeds was explored to evaluate whether the overall genome diversity within and across populations were consistent with expectations based on the origin of the samples and known population relationships from the literature. Detailed explorations followed with focus on dissecting histories of artificial selection for different purposes and past admixture events in shaping this variation. These involve both genome‐wide analyses of known domestication and breed‐defining traits as well as per locus explorations to reveal potentially common origins of alleles underlying shared phenotypes.

### 3.1 Evaluations of the genomic characteristics

From the whole‐genome re‐sequencing data, individual genotypes were called at 50,687,269 loci across the genome. After filtering, 30,617,622 bi‐allelic SNVs were kept. These were first used to perform a comprehensive genome‐wide analysis to explore the overall relationships between individuals within and across the sampled populations as described in the next sections.

#### 3.1.1 The overall population structure described by a principal component analysis

**FIGURE 2:**
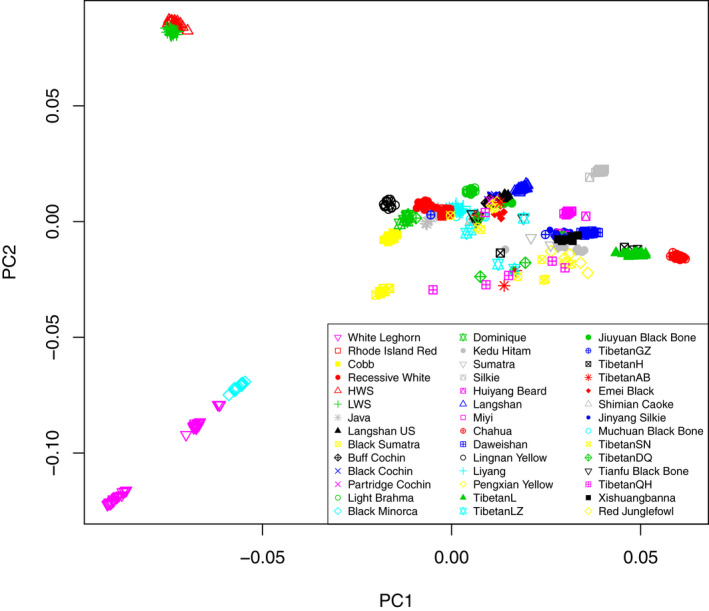
Principal component analysis of the sampled chicken breeds. For all the chicken breeds except the GJF, the first two PCs were plotted and the label for each population was shown in the legend at the bottom left

A principal component analysis (PCA) was used to evaluate the overall population structure across the samples (Figure 2). The first two PC’s captured 67.0% and 7.2% of the variance, respectively. The clustering in the PCA analysis of the genome‐wide SNP data illustrated the close relationship between all but four of the sampled populations. The outliers were two selection lines from an experimental White Plymouth Rock population (the Virginia body weight lines: HWS and LWS). These originate from the same White Plymouth Rock (WPR) population in 1957 and have since been subjected to bidirectional, single‐trait selection in closed populations for either high‐ (HWS) or low‐ (LWS) 56‐day weights (Dunnington et al., 2013; Marquez et al., 2010; Siegel, 1962). The other two populations were the most specialized layer populations included in the sample (White Leghorn (WL) and Black Minorca (BM)). The clear separation of HWS, LWS, WL, and BM from the other populations, as primarily captured by PC1, was not entirely expected. The divergence of the WL from the other breeds was more expected both given its Mediterranean origin and that it has been intensely selected on primarily egg‐laying capacity (Lyimo et al., 2014; Moiseyeva et al., 2003). The clustering of BM with WL is also consistent with BM being used in modern efforts to increase the size of the Leghorn breed (Roberts, 2009). The HWS and LWS are, however, meat‐type birds bred from the heavy White Plymouth Rock but the clustering might in part be due to the finding in an earlier study that the BM was one of the ancestors contributing to the WPR founder population of the HWS/LWS (Guo et al., 2019).

The individuals of the populations sampled from Tibet and Sichuan (China) were often scattered both between, and sometimes also within, the different sampled stocks. This is likely due to the common admixture events in these regions. Close relationships were sometimes also observed between breeds sampled from different geographic regions. An interesting one is between the Chinese breed Liyang (considered a descendent of the original “Shanghai chicken”) and the Rhode Island Red (RIR). These as historical records have reported Chinese breeds from this region being important during the creation of RIR (https://livestockconservancy.org/index.php/heritage/internal/rired).

#### 3.1.2 Model‐based clustering further dissects admixture events implicated in the PCA analysis

**FIGURE 3:**
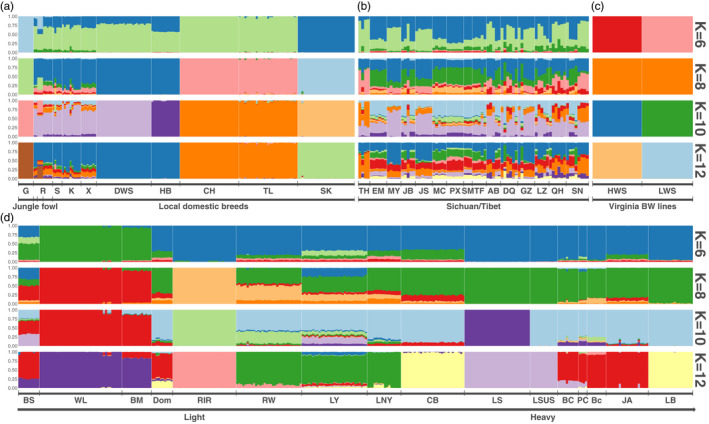
The genomes of chicken breeds worldwide are influenced by old and recent admixture events. Admixture results for four different *K* values: 6, 8, 10, and 12 (top to bottom) are reported. In the plot, each individual is represented by a thin vertical line partitioned into estimated fractions of genome from each cluster (*K*; *y*‐axis). For each *K*, the result was spilt (plotted) into four subgroups (subfigures) which includes junglefowl and local domestic breeds (a), breeds from Sichuan/Tibet (b), Virginia BW lines (c), and highly domesticated breeds with body weight varying from light to heavy (d). The first black line under the plot divides the samples into breeds and the second indicates which group the breeds belong to. Green and Red Junglefowl are labeled as G and R. The Red Junglefowl samples are sorted from left to right based on their sampling sites (Solok, West Java and Yunnan). The abbreviations for the breeds are provided in Table S1

Intentional, or unintentional, cross‐breeding is a central process throughout the histories of our domesticated animals. To evaluate the potential effects of these on the genomes of the sampled populations, a model‐based clustering analysis (Figure 3) was used. In this way, we could explore the admixture events and provide additional insights to the population structure indicated by the PCA analysis (Figure 2). The results are presented by grouping breeds based on either on their origins (wild or local domestic populations) or primary characteristics (specialized populations). A *K* value of 8 was selected based on its minimal cross‐validation error rate, and results from *K* = 6, 8, 10, 12 are presented in Figure 3.

The admixture analyses for the wild ancestors of the domestic chicken and the less intensely selected indigenous Chinese breeds were illustrated in Figure 3a. The Green Junglefowl falls, as expected, into an own cluster in all analyses. Unexpectedly, the Red Junglefowl population sampled in Indonesia has a significant Green Junglefowl introgression, suggesting admixtures between the two Junglefowl species in this area. Overall, however, the Red Junglefowl is very similar—both in terms of ancestry components and proportions—with three of the sampled indigenous breeds: Sumatra (S), Kedu Hitam (KH), and Xishuangbanna (X). A considerable similarity of the population structure was also seen in the 15 indigenous Chinese breeds sampled from the Sichuan/Tibet area. Also, the admixture revealed that the Tibetan breeds are highly admixed (Figure 3b) and are overall closely related to the sampled Red Junglefowl populations (Figure 3a).

The clustering of the more agriculturally developed breeds is shown in Figure 3d. These are sorted with low body weight breeds on the left and high body weight breeds on the right. Overall, they cluster separately from less selected breeds and the junglefowl (Figure 3). This likely reflects large changes in the genome resulting from intensive breeding. The modern heavy US breeds shared common ancestors with breeds from Asia, which was as expected based on historical records and more recent analyses (Dana et al., 2011; Ekarius, 2007; Guo et al., 2019) describing that the Asian chicken breeds have been used to improve the local breeds in the United States. Liyang (a descendent of the Shanghai chicken) is related to multiple Asian and Western breeds further supporting the expected role of this heavy breed, or closely related ancestors of it, in the cross‐breeding (admixture) that were central for producing western meat‐type chickens (Figure 3d). Furthest to the left are light and highly fertile breeds (Figure 3d). One is the modern layer breed White Leghorn, which is distinct from most other breeds except the Black Minorca, a breed described to have been introgressed into the original Leghorn breed to increase its size (Roberts, 2009).

In the heavy body weight group, the Dominique (Dom), an old American multi‐purpose breed that in this analysis, was found to be related to many of the heavy Asian and Western chicken breeds, in particular the Java (JA), Cochins (PC, Bc, and BC), and Light Brahma (LB). The Recessive White (RW), Liyang (LY), and Lingnan Yellow (LNY) are closely related, with only minor differences in contributions from different ancestry sources. The Cobb (CB) is a modern western broiler breed and it shares a majority of ancestry sources with the other heavy breeds. As expected, the Langshan populations from the United States (LSUS) and China (LS) are closely related. Also, the different varieties of the Cochin breed are closely related to each other and with Java.

#### 3.1.3 Extent of linkage disequilibrium (LD) and autozygosity of the genome reflect differences in population histories

By evaluating how the allelic variations across the SNVs are distributed in the genomes of the individual populations, additional insights can be gained into the demographic events that have formed them. To this end, genome‐wide, pairwise LD patterns were estimated within each of the sampled populations (Figure S1) using *PopLDdecay* 3.31. Overall, the results were consistent with expectations given what is known about them regarding effective population sizes, when they were founded and intensity of artificial selection applied. Populations with slow LD decay were generally found to have been founded more recently, and often also more intensely selected in smaller populations. Most extreme are the Virginia BW lines (Dunnington et al., 2013; Marquez et al., 2010; Siegel, 1962), a population of two closed lines that at the time of sampling had been divergently selected for 40 generations with N_e_~35 (Marquez et al., 2010) for a single trait (eight‐week body weight). The most rapid LD decay was observed in older, local breeds from Asia (Figure S1). The realized LD decays thus conformed well with expectations (Vos et al., 2017; Zhang et al., 2019) given the population history of the breeds.

**FIGURE 4:**
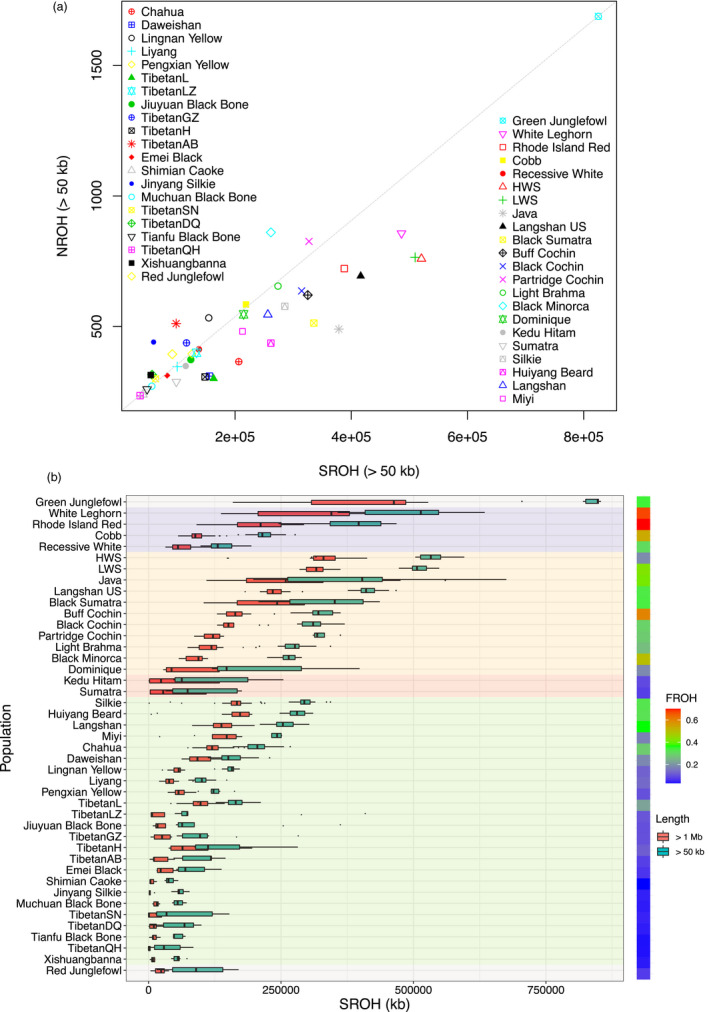
Distributions of numbers and total lengths of runs of homozygosity reflect the demographic history of the chicken populations. (a) The mean numbers (NROH) and total length (SROH) of the ROH are plotted for each population. (b) The box plot presents the total length of long runs of homozygosity (ROH) with runs longer than 50 kb or 1 Mb plotted on the left. The breeds are grouped according to the shadowing as Junglefowls in gray, commercial lines in purple, US breeds in yellow, and breeds from Indonesia/China in pink/green, respectively. The FROH, which captures the mean of the individual coefficient of inbreeding and is calculated using the mean SROH (individual length >50 kb) divided by the reference genome size, is provided for each breed on the right

Autozygosity regions in the genome are stretches of homozygous DNA possibly inherited from a recent common ancestor (Gibson et al., 2006). The number, length, and genomic distribution of such runs of homozygosity (ROH) reflect the demographic history of a population (Jalkh et al., 2015; Kirin et al., 2010). Here, the Green Junglefowl shows the largest sum of ROH (SROH; Figure 4), with most of the individual ROH being short (<500 kb; Table S2). This is consistent with it being a wild, and since long outbred population, that has gone through a bottleneck and subsequent breeding within a small population (Table S2). Another population displaying a large SROH is the experimental Virginia BW lines. This is also consistent with them passing through a bottleneck when forming the base population of the selection experiment and then being subjected to intensive single‐trait selection for many years in closed high‐ and low‐weight selected lines. In this population, the ROH segments are longer, likely due to selection rapidly driving haplotypes carrying selected alleles to fixation (Figure 4b).

When comparing the modern breeds from the United States with the local breeds from China, they generally show a higher autozygosity (Figure 4). This is consistent with the US breeds being founded from a smaller number of chickens from breeds imported from other continents. It is further supported by the observed differences between the Langshan populations sampled in the United States and China. The Chinese local breeds do, in general, have relatively low genomic autozygosity, likely due to lack of strong selection and possibly also few bottlenecks and breeding with larger effective population sizes. The commercial lines vary in their ROH distributions (Figure 4), suggesting that they have likely been subjected to different types of founder events and breeding strategies in the past.

### 3.2 Analyses to disentangle the evolutionary relationships between the populations

The results from the genome‐wide analyses presented above, together with available historical descriptions about breed origins and relationships, are a foundation for evaluating more specific relationships between breed groups and individual populations in more detail. In the sections below, the results from phylogenetic and gene‐centered analyses aiming to provide infer such relationships are reported.

#### 3.2.1 Phylogenetic analyses confirm known and suggest new, connections between populations

**FIGURE 5:**
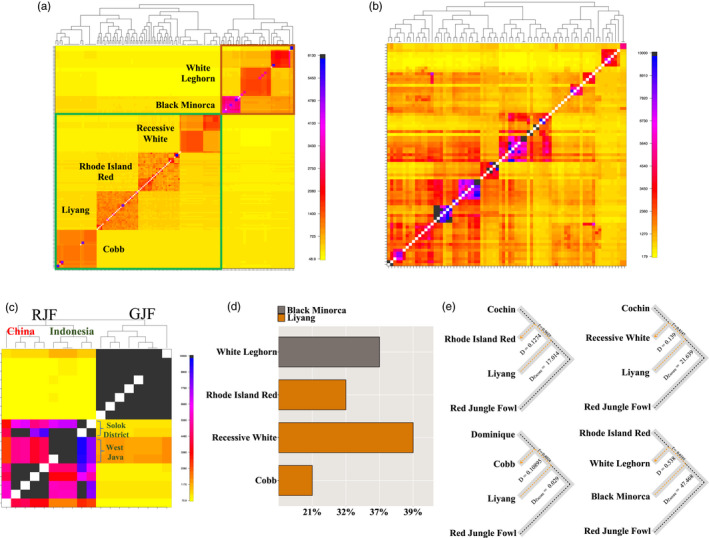
Coancestries between chicken populations revealed by haplotype sharing. Visualizations of coancestry matrixes from a *Finestructure* clustering analysis illustrate the population structure and traces of recent population admixture events in the genomes of related chicken populations. The color in the heat map corresponds to the number of genomic motifs copied from a donor genome (row) to a recipient genome (column). The meat‐type (green box) and egg‐type (brown box) group of breeds (a), the breeds from Tibet and Sichuan (b), and the Junglefowls (c) were plotted. The top ancestry component was plotted for the commercial breeds (d), and graphical representation of clade arrangements for tests of introgression using Patterson's D statistic is provided (e)

A *Finestructure* (Lawson et al., 2012) analysis was used to refine the clustering of the breeds. It estimates the relationships between local breeds from Asia, and the modern breeds used in commercial poultry meat and egg production (Figure 5a). Modern breed cluster together to confirm known—or suggest new—relationships between these.

The genome‐wide haplotype sharing, as estimated by the chunk count matrix from *Finestructure*, overall highlights that sharing is largest between the closest clustering populations (Figure 5a–c). For example, the heavy (meat‐type) and light (egg‐type) breeds cluster separately in the analysis and the strongest relationships are found among samples from the same populations (Figure 5a). The likely admixture events among the indigenous breeds from Tibet and Sichuan result in close relationships among these populations also in this haplotype‐based analysis (Figure 5b). Some particular features, however, deserve to be highlighted. There is a subgroup difference in the contribution by the RJF (Figure 5c). This likely reflects a continental difference between the RJF individuals used, where some were from southern China and others from Indonesia. Despite the sampling of RJFs from West Java and Solok districts in Indonesia from nearby geographic locations, they still appear different. Figure 5c highlights that the RJF population from West Java contributed to the GJFs, suggesting admixture events between these populations in the past.

Phylogenetic trees were computed to further disentangle population relationships. Overall, they confirmed expected relationships between the populations based on historical records and their geographic origins (Figure S2). Some particularly interesting and/or unexpected relationships were, however, revealed. These include those between (i) Liyang and Rhode Island Red, confirming records that Shanghai chickens were used to develop the RIR breed in the United States and (ii) White Leghorn and Black Minorca, a relationship described in the historical records but not reported before in genomic studies.

#### 3.2.2 Explicit testing of breed admixture events and gene flow between them

Consistent with available historical records (Roberts, 2009), the genomic analyses above identify a genetic relationship between the Black Minorca and White Leghorn breeds (Figures 2, 3 and 5a). These breeds also display phenotypic resemblances including white earlobes, good egg production, and white eggshells. Using *Globetrotter* (Hellenthal et al., 2014), we quantified the relationship between these breeds. The Black Minorca was identified as a major ancestor of the White Leghorn (Figure 5d). Patterson's D statistic (Green et al., 2010) was used to test the genome‐wide excess of shared derived alleles between these breeds and estimate the proportion of genomes shared. A trio of another modern layer (RIR), White Leghorn and Black Minorca, was analyzed using Red Junglefowl as outgroup (Figure 5e). The estimated proportion of shared derived alleles from the Black Minorca to the White Leghorn was 61.6% (significantly positive *D* = 0.54; *D*_Zvalue_ = 47.5).

The Rhode Island Red (RIR) breed was originally developed as a dual‐purpose breed and was later more intensely selected for use as a specialized layer breed. Historical records describe that the RIR originates from crosses of Cochin, Java, Malay, Shanghai, and brown Leghorns (Ekarius, 2007). Here, a trio of Cochins, RIR, and Liyang were analyzed, where the Liyang was used as a proxy for the Shanghai chicken as it is a modern descendant of this stock (Chen et al., 2011). A large (56.3%) and significant (significantly positive *D* = 0.13; *D*_Zvalue_ = 17.0; Figure 4e) contribution by Liyang to RIR was revealed in this analysis.

#### 3.2.3 Selection signatures in the genome and exploration of candidate pigmentation genes

Pigmentation traits are highly selected in domestic populations and often a distinguishing feature between breeds. Comparisons of *F*_st_ between recessive white and black populations and haplotype selection identified a selection signal at the *TYR* (Figure S3A,C) gene region (Tobita‐Teramoto et al., 2000). This was consistent with earlier reports of it being the gene causing recessive white feathering in chickens. A common haplotype was fixed in the evaluated recessive white populations including Silkie, Recessive White, and White Plymouth Rock (represented by the Virginia BW lines) (Figure S3D). A fraction of the individuals in dominant white breeds were also found to carry a haplotype at the recessive white locus that is closely related to the one found in the recessive white breeds (Figure S3D). This finding is consistent with a previous report describing historical crosses between dominant and recessive whites (Knox, 1927) where Silkie was found to carry the recessive white allele.

Dominant white was defined early in poultry and one of the first traits used to verify Mendel´s laws in chicken (Bateson & Saunders, 1902). Here, the breeds described as dominant white (White Leghorn and the Cobb from two different grandparental lines A and D) are expected to carry both the dominant white (*I*) and extension (*E*) alleles. Selective sweep analyses to evaluate the basis for the dominant white was performed by comparing dominant white/recessive white and dominant white/black breeds, and both identified the strongest signal in the region of the *ERBB3* gene (Figure S4A,B,D). This is a surprising observation as another gene, the *PMEL17*, has earlier been reported as causing the dominant white trait in chicken (Kerje et al., 2004). The *ERBB3* encodes a member of the epidermal growth factor receptor (*EGFR*) family of receptor tyrosine kinases. It is known to be located in the region associated with the dominant white phenotype in several linkage mapping studies (Kerje, Carlborg et al., 2003; Kerje et al., 2004; Ruyter‐Spira et al., 1997).

Black birds carry the *E* allele permitting extension of color to all parts of the plumage, while yellow birds carry the *e* allele (Dunn, 1922). Comparisons between the yellow and black breeds in this study detected a signal on the *MC1R* gene (Figure S5A,D) with fixation for alternative haplotypes in them (Figure S5D). This is consistent with earlier reports of *MC1R* being the causal gene at this locus (Kerje, Lind et al., 2003). As white plumage chickens are unable to display the genetic effects of alleles at other coloring and patterning loci, it was not possible to distinguish between recessive/dominant white and black at the *E* locus in this study (Figure S5B,C).

## 4 DISCUSSION

There is an extensive phenotypic diversity among domestic chicken breeds. Consistent with the proposed commensal pathway of domestication (Larson & Burger, 2013; Vigne, 2011) examples can be found in the worldwide chicken populations of breeds being at various stages of this model. Populations at the early stages of habituation, commensalism, and partnership can be found among indigenous, local chicken populations. These exhibit primary adaptations related to behavioral and physiological needs for living near humans and in new climates such as high altitudes. Other populations have been raised in captivity and intensely bred for hundreds of years and developed, for example, extreme fancy and agricultural traits.

During above process, admixture events have played a central role in shaping the genomic variation of modern livestock (Decker et al., 2014; Larson & Burger, 2013). Cross‐breeding has been implemented both to develop new and improve existing, chicken breeds for more efficient food production (Guo et al., 2019) as well as to utilize heterosis for improved production. Most important chicken breeds and populations of today have a relatively short history, rarely exceeding a few hundred generations, and these often involve admixtures of several breeds. For some, written documentation about the origin of central breeds exists but to gain insights to the relative contributions by each founder populations more work is needed. Further, the historical sources do not always agree on which breeds were actually used. Recently, a work showed for the White Plymouth Rock breed how genomics could help both differentiating between alternative hypotheses about its origin and to quantify the contributions by each founder breed (Guo et al., 2019). Here, we used whole‐genome re‐sequencing data to explore the admixture events involving Asian chicken breeds after the wave of imports of such breeding stock to Europe and the US aiming to improve the local stocks for more efficient food production (Burnham, 1855). This by screening the genomes for traces of these admixture events, and the subsequent intense artificial selection in the newly formed populations, in modern breeds to evaluate their role in providing genetic variation facilitating the current high levels of performance in the poultry industry.

### 4.1 Admixture events between diverse populations have shaped the genomes of modern chicken breeds

The importance of population (breed) admixture in the later stages of intensive breeding and commercialization of the chicken for food production was explored in detail. Overall, the results show a major contribution by Asian populations to the worldwide chicken population consistent with several waves of introgression followed by population admixture and continued selection. Several detailed findings, where genomics either confirms proposed introgressions or suggest new possible origins of important current breeds, were revealed. Genomic evidence suggests a large contribution by the Shanghai chicken, here represented by the modern descendent the Liyang breed, to major breeds used during the establishment of modern commercial broiler stocks: the Rhode Island Red and Recessive White. The findings here add to earlier work (Dana et al., 2011; Ekarius, 2007; Guo et al., 2019) emphasized the contribution by Asian chicken breeds to the genetic variation utilized in the chicken industry.

Except the improvement by the large Asian breeds, the Mediterranean fertile stock Black Minorca also play an important role in modern layer breeding. Evidence from both the historical records (Roberts, 2009) and our genomic inference have clarified the admixture of Black Minorca and White Leghorn. The White Leghorn is a representative of the Mediterranean breeds, which in earlier studies were found to be genetically distant from many Asian breeds (Lyimo et al., 2014; Malomane et al., 2019). Similar results were found here in the PCA analysis, with the White Leghorn/Black Minorca cluster being quite distant from all other Asian breeds. The Mediterranean breeds are egg‐type breeds with distinguishing features being white earlobes, laying of white eggs, being broody and able to tolerate extreme heat (Hutt, 1938; Lamoreux & Hutt, 1939). Further work is needed to disentangle the detailed genealogy and development of this egg‐laying breed cluster, but it appears likely that a combination of intense selection for a different group of traits combined with starting from a different selection of locally adapted ancestral breeds a significant time ago would have contributed to their divergence from the heavier Asian and western chicken breeds.

### 4.2 Homozygozity patterns likely influenced by the breeding history

The ROH distribution was used to investigate the population demographic history. An increased burden of ROH was common in the analyzed US samples, likely a reflection of a limited effective population size in the past and recent inbreeding. A different pattern was observed for the Virginia BW lines and the White Leghorn with enrichments in both long ROH and the total sum ROH (Figure 4; Table S2). This suggests both ancient and recent inbreeding (bottlenecks) as well as intense selection happened in the breeding history.

### 4.3 Population substructure of junglefowls reflecting their geographic origin

Subgroups were identified in the Red Junglefowl population (Figure 5c). The structures in these are consistent with the sampling locations, the RJFs were sampled from west Java, Solok district (Indonesia) and Yunnan (China) (Guo et al., 2016; Ulfah et al., 2016; Wang et al., 2015). In the RJF, the structure suggests multiple ancestry populations (Figure 3a). This is likely due to the sampled birds not being pure but rather admixed with domestic chickens at some point in history as suggested by the close relationship between RJFs from China and the Chahua breed (Chen et al., 2011) as well as the RJFs from Java with the GJF (Figure 5c).

## 5 CONCLUSIONS

Here, we describe the population structure of worldwide chicken breeds and report the most comprehensive genome‐wide comparison between local Asian and modern Western chicken breeds to date. The genome‐wide relationships between the populations were explored to confirm suggested and suggest novel relationships between Asian, Western, and Mediterranean breeds, as well as molecular genetic mechanisms of breed‐defining phenotypes. A large contribution by Asian chicken breeds to Western breeds were found via the introgression of locally developed breeds, illustrating the value of utilizing worldwide genetic variation for the progress of modern poultry breeding.

## CONFLICT OF INTEREST

The authors declare that there is no conflict of interest.

## Supporting information

Supplementary Material

## Data Availability

The raw sequence data for this study are available at NCBI with BioProject numbers PRJNA597842, PRJNA547951, and PRJNA552722.
