# Supplementary material for: Researching on the fine structure and admixture of the worldwide chicken population reveal connections between populations and important events in breeding history
Source: Evol Appl. 2021 May 5;15(4):553–64. doi: 10.1111/eva.13241 (PMC9046761; doi:10.1111/eva.13241)
Supplement: Supplementary file 1 — Supplementary Material [file EVA-15-553-s001.pdf]

Supplementary Material

Supplementary Figure 1. Evaluation of the LD decay across the sampled world-wide chicken populations. The decay of LD was plotted as pair-wise r^2 values against the physical distance in kb.

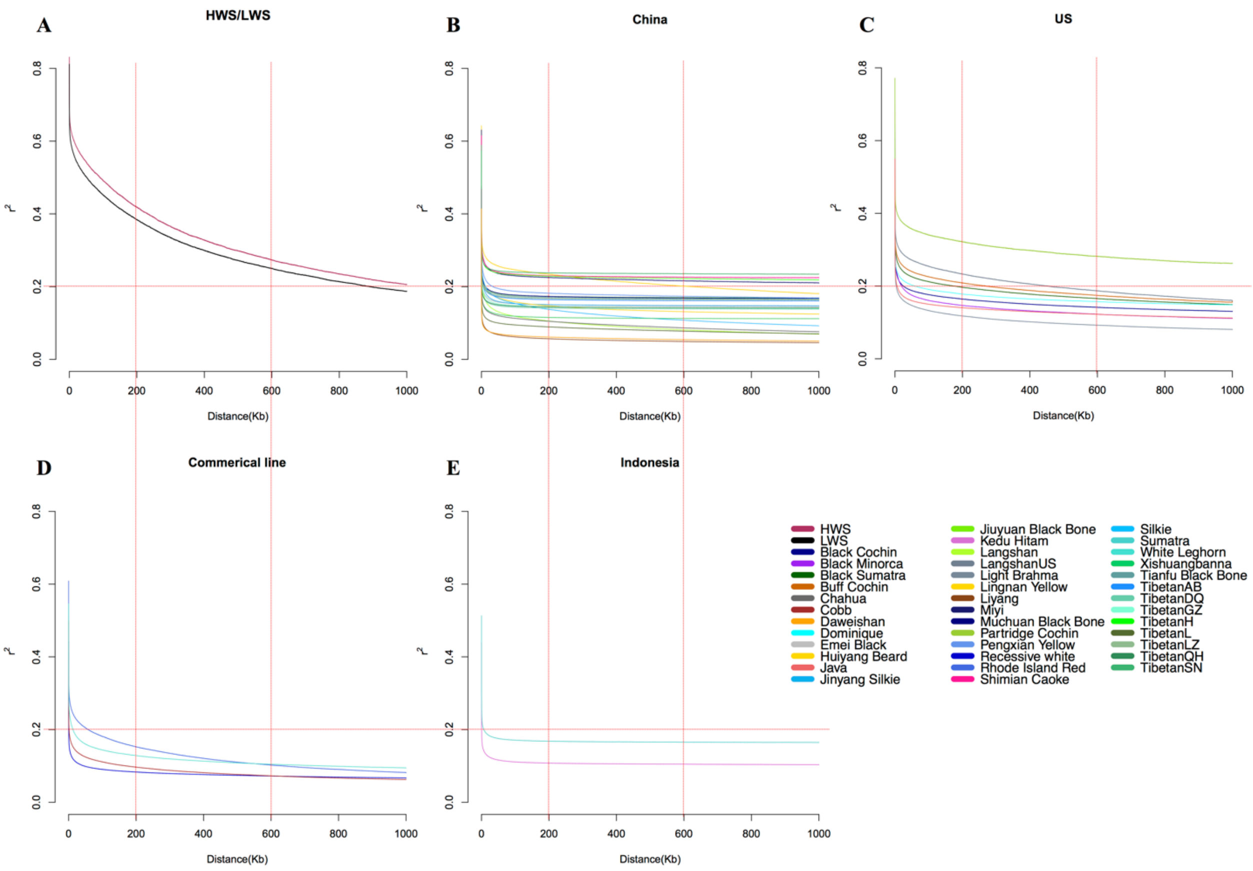

Supplementary Figure 2. Population dendrogram for the studied sample of world-wide chicken populations. The population dendrogram was plotted using the genetic relatedness matrix. The coloured backgrounds are used to separate the populations into related groups of breeds as indicated in the legend. The population branches at the bottom are from the Sichuan and Tibetan regions in China. Darker pink colour is used for some of the populations in the group of Chinese indigenous breeds to represent samples of these from the US.

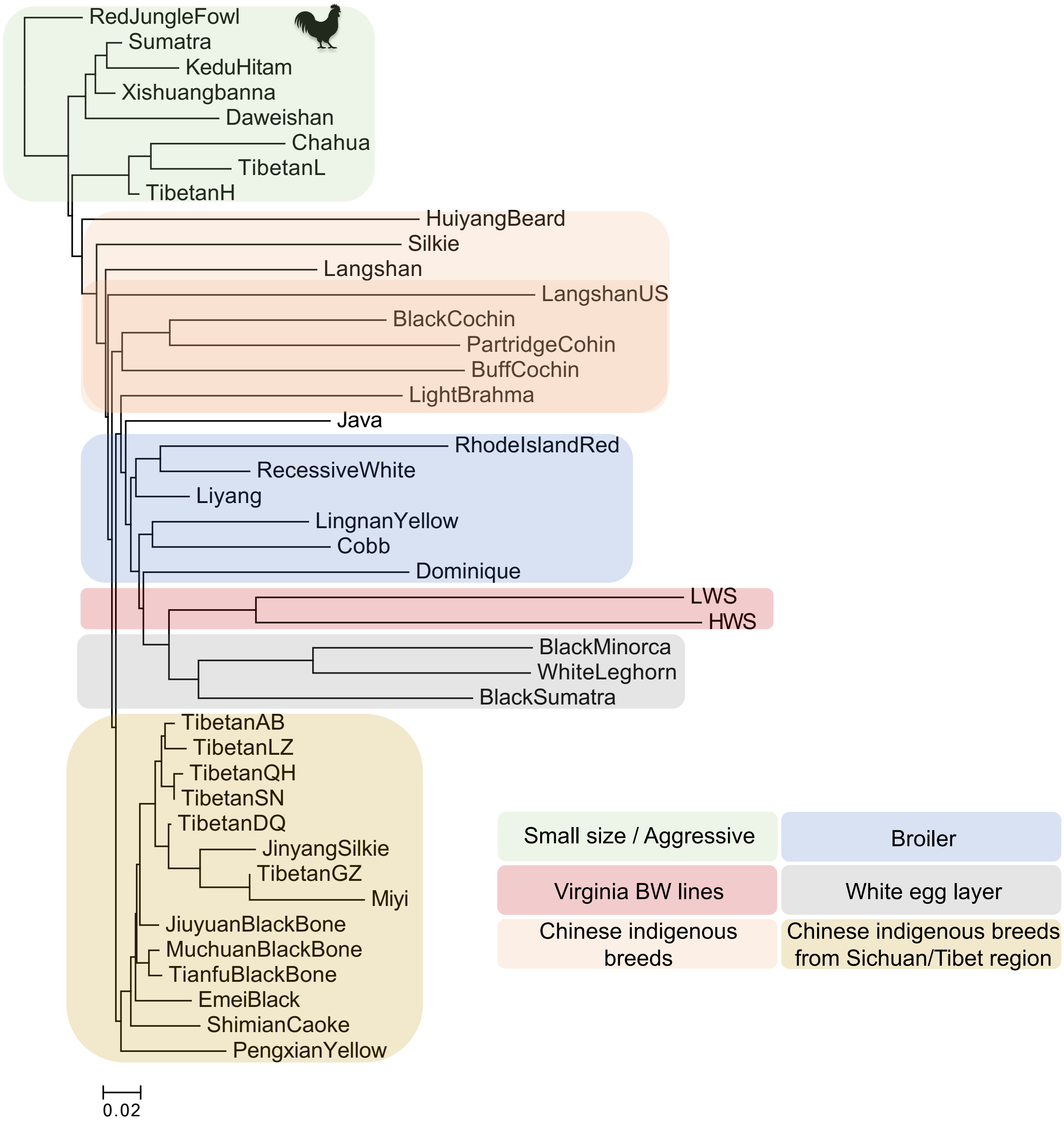

Supplementary Figure 3. Detection of selection signatures and haplotypes at the recessive white locus on chromosome 1. F_{st} screening at the recessive white locus between black birds and recessive white (**A**) / dominant white (**B**) birds. Genes located in the high F_{st} regions are listed. (**C**) Signatures of selection over the *TYR* gene region revealed by hapFLK analysis. (**D**) Illustration of the haplotypes at the recessive white locus in recessive and dominant white birds. Yellow arrows point the haplotypes in the dominant white individuals that are in concordance with the recessive white ones. Brown bar at the bottom indicates the *TYR* gene region.

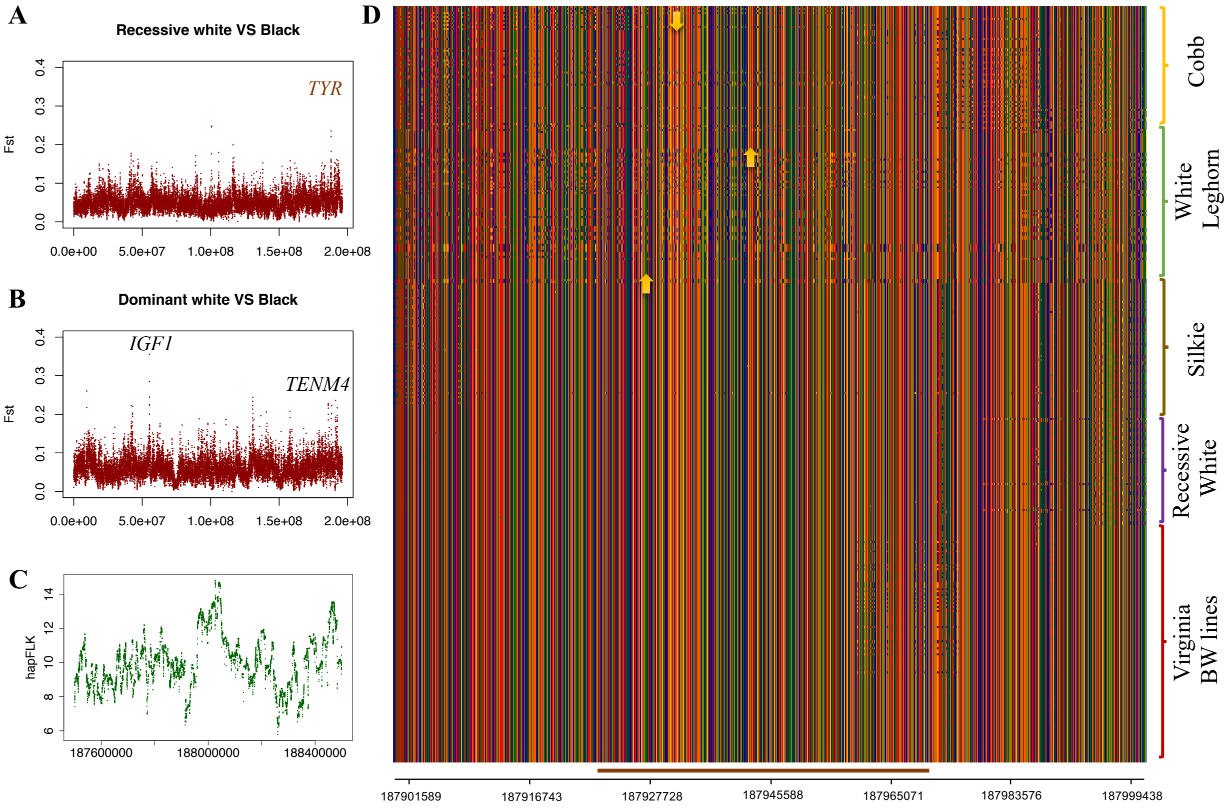

Supplementary Figure 4. Detection of selection signatures and haplotypes at the dominant white locus on chromosome 33. F_{st} screening at the dominant white locus contrasting dominant vs. recessive white birds (**A**), dominant white vs. black birds (**B**), and between recessive white vs. black birds (**C**). Genes located in the high F_{st} regions are listed. (**D**) Signatures of selection over the target region from 600-700 kb on chromosome 33 identified by the *hapFLK* analysis. The haplotypes at *ERBB3* (**E**) and *PMEL17* (**F**) loci for both dominant and recessive white birds are illustrated. Brown bar at the bottom show the gene region.

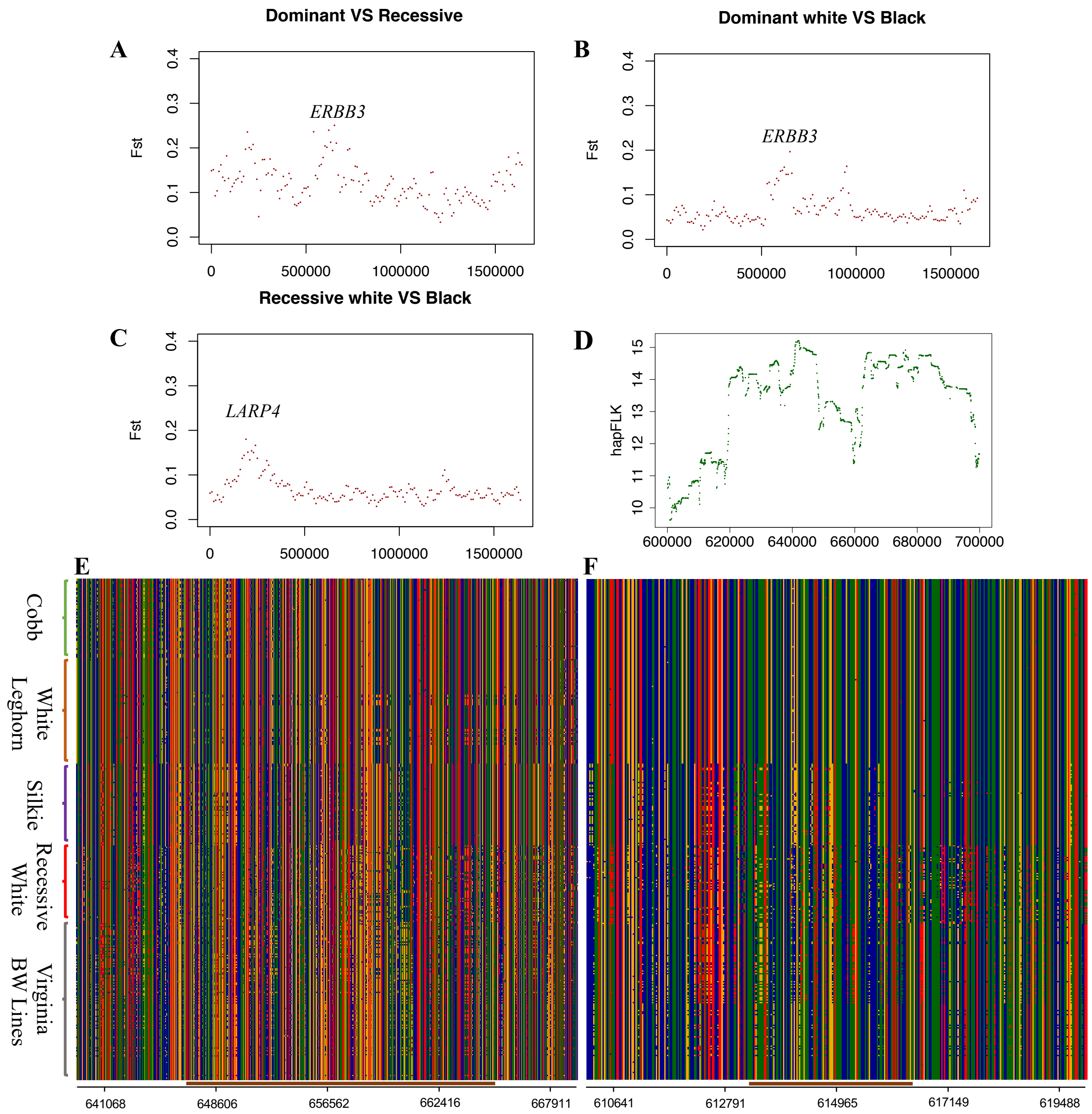

Supplementary Figure 5. Detection of selection signatures at the *MC1R* locus on chromosome 11. F_{st} screening at the *MC1R* locus between yellow and black feathered birds with (**A**), reporting the screen between recessive white and black feathered populations (**B**), and between dominant and recessive white populations in (**C**). Genes located in the high F_{st} regions are listed. In (**D**), the selection signal at the *MC1R* region based on *hapFLK* analysis is shown and in (**E**), the haplotypes at the *MC1R* locus in yellow and black birds are given. The brown bar at the bottom illustrates the gene region.

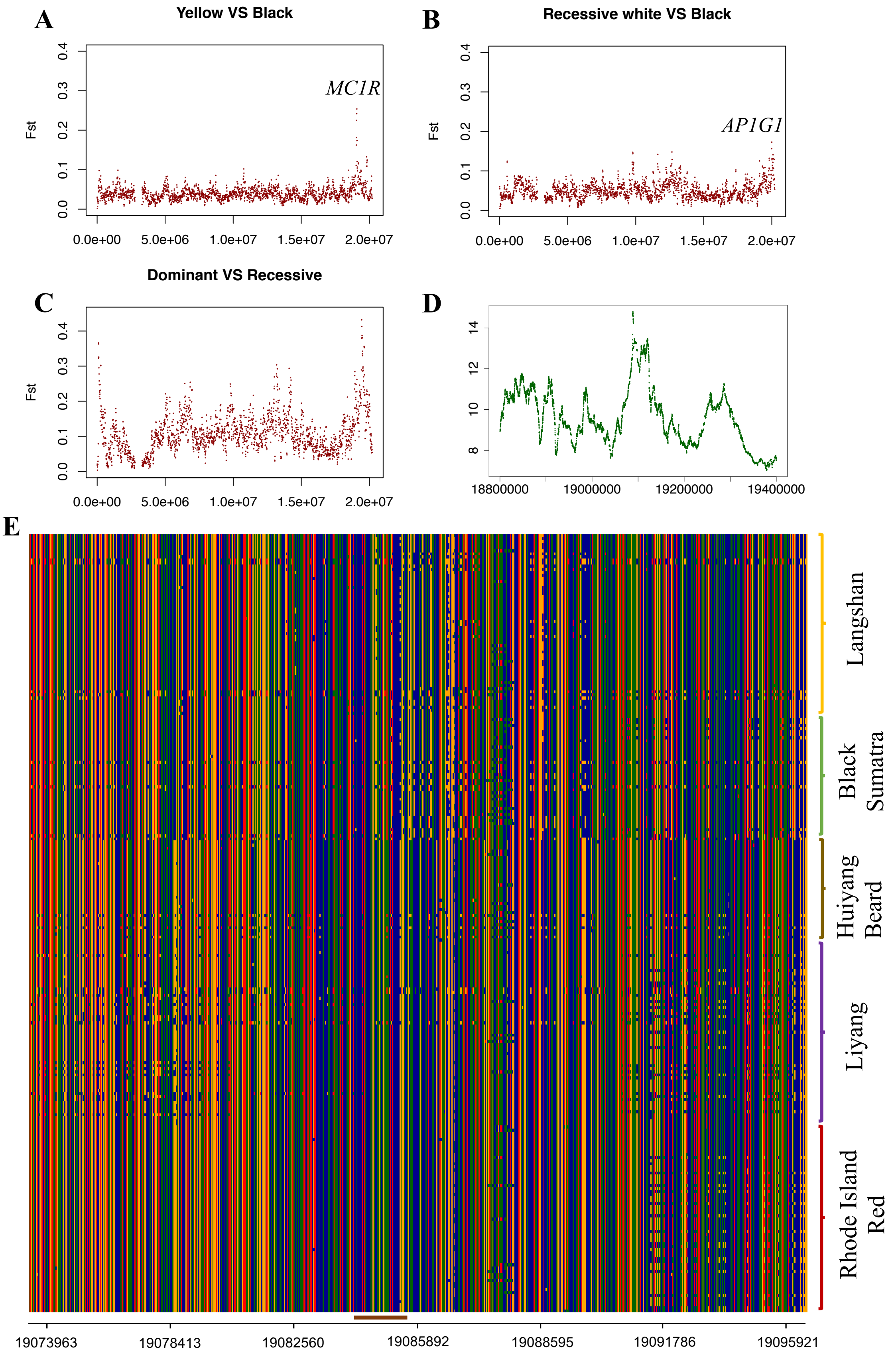
A
Yellow VS Black
MC1R
Fst
B
Recessive white VS Black
AP1G1
Fst
C
Dominant VS Recessive
Fst
D
E
Langshan
Black Sumatra
Huiyang Beard
Liyang
Rhode Island Red
19073963
19078413
19082560
19085892
19088595
19091786
19095921

Table S1. Breed information, including origins of samples, names and sample sizes.

**Population**	**Abbreviation**	**Samples**	**Origin**	**Sub-location**	Source of data
Virginia high body weight line	HWS	29	US	Virginia	(Guo et al. 2019)
Virginia low body weight line	LWS	30	US	Virginia	
Black Cochin	BC	10	US	-	
Black Minorca	BM	14	US	-	
Black Sumatra	BS	10	US	-	
Buff Cochin	Bc	9	US	-	
Langshan US	LSUS	13	US	-	
Light Brahma	LB	21	US	-	
Dominique	Dom	10	US	-	
Partridge Cochin	PC	4	US	-	
Cobb	CB	30	US	-	Current study
Recessive White	RW	31	US	-	
Rhode Island Red	RIR	30	US	-	
White Leghorn	WL	39	Itlay	-	36 from current study and 3 from (Ulfah et al. 2016)
Java	JA	10	US	-	(Guo et al. 2019)
		10	Indonisia	Java	(Ulfah et al. 2016)
Kedu Hitam	KH	10	Indonisia	Kedu Hitman	(Ulfah et al. 2016)
Sumatra	S	5	Indonisia	Sumatra	
Green Junglefowl	G(RJ)	2	Indonisia	Java	
		6		Madura	
Red Junglefowl	R(JF)	4	China	Yunan	(Wang et al. 2015)
		5	Indonisia	West Java/Solok	(Ulfah et al. 2016)
		1	China	Yunan	(Nie et al. 2016)
Chahua	CH	31	China	Yunnan	Current study
Daweishan	DWS	29	China	Yunnan	
Huiyang Beard	HB	15	China	Huiyang	

Langshan	LS	31	China	Yangzhou	
Lingnan Yellow	LNY	16	China	Guangzhou	
Liyang	LY	31	China	Jiangsu	
Silkie	SK	30	China	Jiangsu	
Xishuangbanna	X	8	China	Yunnan	(Nie et al. 2016)
Emei Black	EM	6	China	Sichuan	(Nie et al. 2016)
Jinyang Silkie	JS	6	China	Sichuan	
Jiuyuan Black Bone	JB	5	China	Sichuan	
Miyi	MY	5	China	Sichuan	
Muchuan Black Bone	MC	5	China	Sichuan	
Pengxian Yellow	PY	6	China	Sichuan	
Shimian Caoke	SM	3	China	Sichuan	
Tianfu Black Bone	TF	5	China	Sichuan	
TibetanAB	AB	5	China	Aba, Tibet	
TibetanDQ	DQ	6	China	Diqing, Tibet	
TibetanGZ	GZ	6	China	Ganzi, Tibet	
TibetanLZ	LZ	5	China	Lingzhi, Tibet	
TibetanQH	QH	6	China	Qinghai	
TibetanSN	SN	8	China	Shannan, Tibet	
TibetanH	TH	4	China	Tibet	(Wang et al. 2015)
TibetanL	TL	31	China	Yanzhou	Current study

Table S2. The distributions of average NROH of different lengths.

Population	> 50 kb	50-500 kb	> 500 kb	0.5-1 Mb	> 1 Mb	1-2 Mb	> 2 Mb	2-4 Mb	4-8 Mb
HWS	759	455	304	144	160	104	56	46	9
LWS	765	469	295	139	156	103	54	43	10
Black Cochin	703	500	203	105	98	68	30	26	4
Black Minorca	2027	1684	344	218	126	102	24	24	1
Black Sumatra	1034	678	356	145	211	113	98	71	22
Buff Cochin	695	479	215	117	98	67	32	26	5
Chahua	628	428	200	98	102	66	36	29	6
Cobb	1011	800	211	122	89	63	26	23	3
Daweishan	447	345	102	43	59	32	27	20	7
Dominique	596	473	123	72	51	36	15	12	2
Emei Black	313	277	36	18	18	12	6	5	1
Green Junglefowl	1688	1207	481	265	216	152	65	57	7
Huiyang Beard	647	424	223	101	122	76	46	38	7
Java	523	309	215	89	126	71	55	42	12
Jinyang Silkie	440	427	13	11	2	2	1	1	0
Jiuyuan Black Bone	373	318	55	25	30	18	12	9	3
Kedu Hitam	349	305	44	20	24	12	11	7	3
Langshan	1021	779	242	126	116	74	42	33	8
Langshan US	693	446	247	125	122	82	40	34	6
Light Brahma	1027	780	247	143	104	77	27	24	3
Lingnan Yellow	533	458	75	44	31	24	6	5	1
Liyang	589	511	78	45	33	22	11	9	2
Miyi	481	373	108	50	59	37	22	16	5

Muchuan Black Bone	271	249	23	15	8	6	2	2	0
Partridge Cochin	826	634	193	123	70	54	16	15	2
Pengxian Yellow	395	334	61	31	30	20	10	8	2
Recessive White	703	592	111	60	51	35	17	13	3
Red Junglefowl	394	354	40	25	15	12	3	2	1
Rhode Island Red	1253	861	391	206	185	128	58	49	9
Shimian Caoke	239	224	15	11	4	3	1	1	0
Silkie	818	598	220	110	110	71	39	31	7
Sumatra	288	251	37	18	19	11	8	4	3
Tianfu Black Bone	260	242	17	11	6	5	2	1	0
TibetanAB	511	476	35	22	12	8	4	4	0
TibetanDQ	315	296	19	13	6	4	2	2	0
TibetanGZ	490	436	54	31	23	15	8	6	2
TibetanH	307	236	71	31	40	22	18	13	4
TibetanL	517	363	153	72	81	51	30	25	5
TibetanLZ	399	340	59	29	29	18	12	8	3
TibetanQH	329	315	14	11	3	2	0	0	0
TibetanSN	300	275	25	17	8	5	3	2	0
White Leghorn	1542	1089	453	214	240	143	96	74	20
Xishuangbanna	314	297	17	12	5	4	1	1	0
